# The correlation between height and assay parameters in children with short stature

**DOI:** 10.1097/MD.0000000000048883

**Published:** 2026-05-22

**Authors:** Chuanwei Ban, Juan Wang, Qian Zeng, Ping Liu, Qingling Wang, Xin Lv

**Affiliations:** a Clinical Laboratory, Children’s Hospital Affiliated to Shandong University, Jinan, Shandong Province, China; b Clinical Laboratory, Jinan Children’s Hospital, Jinan, Shandong Province, China.

**Keywords:** correlation, height, parameters, short stature

## Abstract

It is known that growth hormone plays a major role in children’s height. This research was conducted to explore the connection between children’s height and other parameters for potential diagnostic markers in cases of short stature. Information was gathered from children diagnosed with short stature from our hospital, including fundamental data and assay findings, and was evaluated for the correlation between height and assay findings. The correlation between height and assay findings was evaluated using the Pearson correlation coefficient test. A total of 2396 children were included, with 61.4% being boys, who exhibited a higher prevalence across most age groups. The findings revealed moderate positive correlations between height and iron, hemoglobin, as well as a weak correlation with zinc (iron, *r* = 0.48, hemoglobin, *r* = 0.52, zinc, *r* = 0.29, *P* < .0001, respectively). Additionally, a moderate negative correlation was observed between height and 25-hydroxyvitamin D (*r* = −0.37, *P* < .0001). Height showed highly positive correlations with insulin-like growth factor-1 (*r* = 0.67, *P* < .0001) and insulin-like growth factor binding protein 3 levels (*r* = 0.66, *P* < .0001), and moderate positive associations with sex hormones (follicle-stimulating hormone: *r* = 0.43, luteinizing hormone: *r* = 0.42; testosterone: *r* = 0.5, *P* < .0001, respectively). Furthermore, a moderate positive correlation was found between height and glucose (*r* = 0.41, *P* < .0001), while no significant correlation was observed between height and lipid metabolism parameters. In summary, short stature is a prevalent issue in our country. The research emphasized the correlation of height with insulin-like growth factor-1, insulin-like growth factor binding protein 3, and other parameters, and a negligible correlation with lipid metabolism parameters. These findings can serve as valuable parameters for parents to seek medical attention for children exhibiting abnormal results.

## 1. Introduction

Throughout the process of children’s development, parents frequently express concern regarding their children’s height, given its implications for their nutrition and overall progress. Height represents a critical metric in evaluating children’s well-being, with insufficient height potentially arising from various factors such as primary or secondary growth disorders or unknown origins.^[1]^ Generally, short stature is defined as a height below 2 standard deviations or below the third percentile compared with the average height of healthy children of similar age, gender, and ethnicity in a comparable setting.^[2,3]^ Recent research in China has revealed that approximately 3.2% of children are affected by short stature, underscoring its prevalence in clinical settings.^[4]^ The consequences of short stature extend beyond physical health to impact mental wellness,^[3,5]^ with certain studies suggesting a connection between short stature and an elevated risk of suicidal ideation.^[6]^ Although growth hormone (GH) is widely recognized as a core factor regulating children’s height,^[7]^ the current clinical and basic research lacks systematic and large-sample empirical evidence on the correlation between children’s height and multiple laboratory assay parameters (including trace elements, 25-hydroxyvitamin D, blood glucose, sex hormones, lipid metabolism indicators, and other parameters). Most relevant studies focus on a single type of indicator rather than a comprehensive analysis,^[8,9]^ which leads to the lack of integrated reference indicators for the early screening and auxiliary diagnosis of short stature. This research gap is the core motivation for us to conduct this large-sample correlation analysis. The aim is to identify potential biomarkers associated with short stature and provide practical guidance for clinicians.

## 2. Materials and methods

### 2.1. Study population

This retrospective study was conducted by collecting data from pediatric patients admitted for the first time to the Children’s Hospital Affiliated to Shandong University due to short stature over a 1-year period (May 1, 2023, to April 30, 2024). The sample size calculation for this study was based on the Pearson correlation coefficient test for univariate correlation analysis, performed using GPower 3.1 software (Franz Faul, Heinrich-Heine-Universität Düsseldorf). Calculation parameters were set as follows: expected correlation coefficient *r* = 0.3 (moderate correlation), significance level α = 0.05 (two-tailed test), and test power 1−β = 0.95. The “Height and weight standardized growth charts for Chinese children and adolescents aged 0 to 18 years” were used as a reference for diagnosing short stature. Inclusion criteria included a body height below 2 standard deviations or under the third percentile when compared with the average height of healthy children of the same age, sex, and race in a similar environment. Exclusion criteria included developmental abnormalities of the skeletal system, such as osteomalacia, severe metabolic disorders, organic diseases, hypothyroidism, or chromosomal abnormalities.

### 2.2. Data collection

The research investigated data from patients who were admitted to the Children’s Hospital Affiliated to Shandong University for the first time due to short stature. The hospital maintains a comprehensive pediatric diagnosis and treatment system. Its laboratory detection platform holds ISO 15189 accreditation for medical laboratory quality and competence, which ensures the accuracy and comparability of assay data in this study. Details gathered from the hospital’s information system included basic information such as gender, age, height, and results of laboratory tests. The atomic absorption method was utilized to identify trace elements (iron [Fe] and zinc [Zn]), 25-hydroxyvitamin D levels were assessed using fluorescence immunochromatography, hemoglobin (Hb) levels were determined through the sodium dodecyl sulfate Hb test, glucose levels were evaluated with the hexokinase assay, and hormone levels were measured via the chemiluminescence technique. Triglyceride (TG) and total cholesterol (TC) levels were analyzed using colorimetry, while lipoprotein cholesterol levels were determined using the direct method. The collected data were analyzed after removing outliers.

### 2.3. Statistical analysis

By conducting a Pearson correlation coefficient test, this research investigated the correlation between different height levels and other variables. The statistical analysis was carried out with the GraphPad Prism (GraphPad Software, Inc.) software, where the *P* < .05 was determined to indicate statistical significance.

## 3. Results

### 3.1. Characteristics of study participants

**Figure 1.**
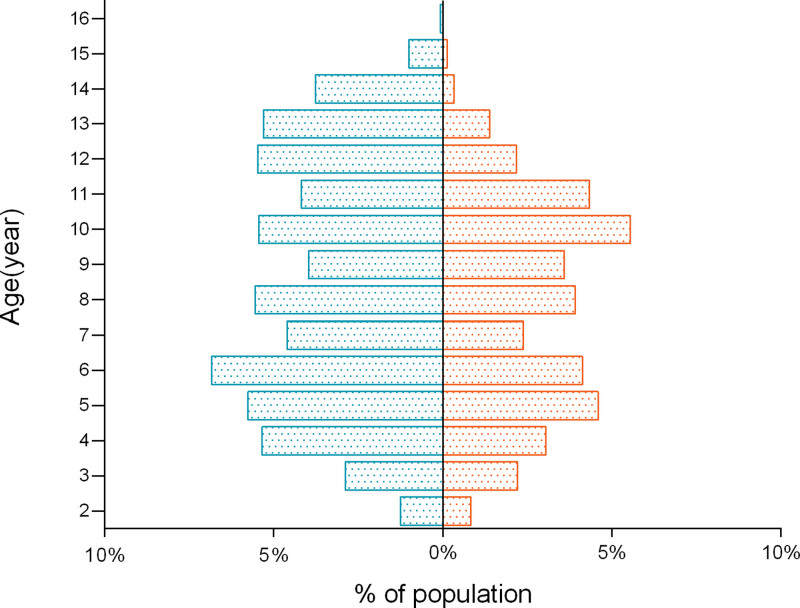
The distribution of participants based on sex and age (the blue bars on the left represent boys and the orange bars on the right represent girls).

**Figure 2.**
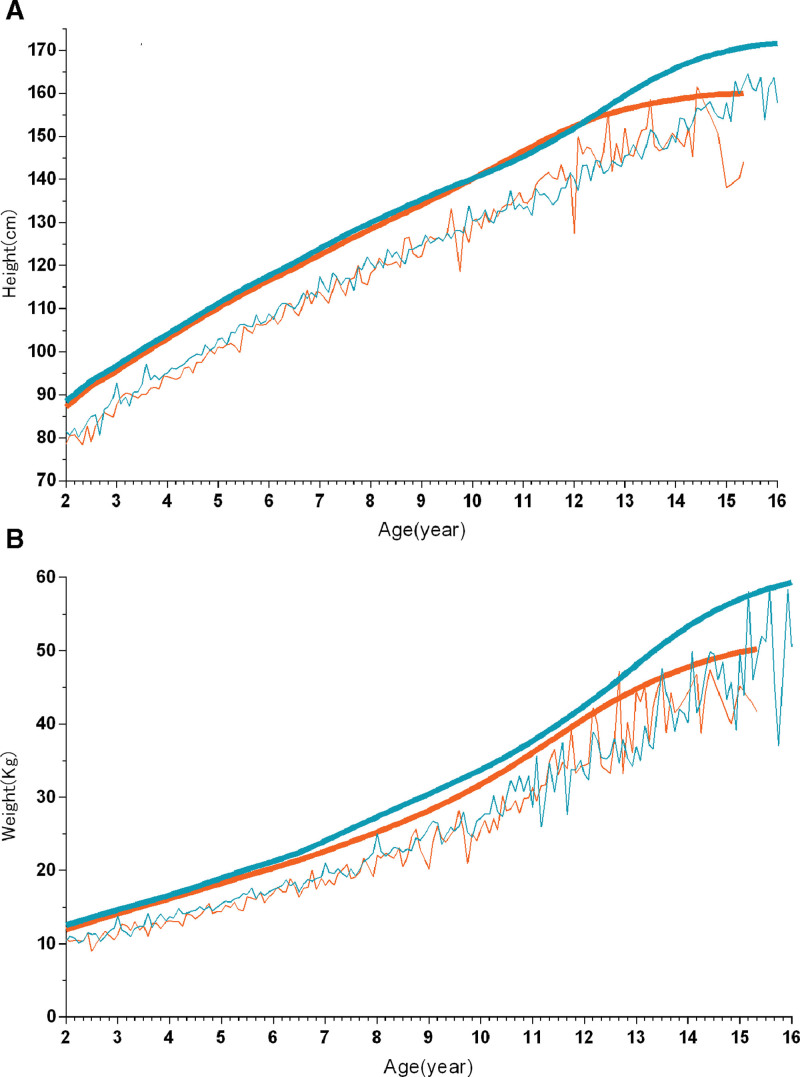
Comparison of the participants’ height (A) and weight (B) with the standard curve. The thick lines represent the standard curves for height (A) and weight (B), while the thin lines indicate patients with short stature. The blue lines represent boys, and the orange lines represent girls.

In total, 2396 children diagnosed with short stature were analyzed (far exceeding the minimum sample requirement of 108 calculated), with a majority of 61.4% being male. The demographic information of the participants is summarized in Table 1. The findings of the different parameters are elaborated in Table 2. We depict the distribution of participants based on sex and age, as shown in Figure 1, and the comparison of participants’ height and weight with the standard curve is shown in Figure 2.

**Table 1:** The participant demographics of the research (n = 2396).

	Boys (n = 1471)		Girls (n = 925)	
	Range	Median	Range	Median
Height (cm)	72.6–169.1	123.2	67.4–161.5	121.1
Weight (kg)	8.4–74	23.2	8.5–58.6	21.8
Father’s height (cm)	150–188	170	150–188	170
Mother’s height (cm)	120–174	157	130.4–175	158

**Table 2:** Laboratory parameters of children with short height (n = 2396).

Parameters	Range	Median	Number of tests
Fe (mmol/L)	6.01–10.26	7.85	995
Zn (μmol/L)	35.6–115.3	68.6	995
25-hydroxyvitamin D (ng/mL)	4.89–90.58	23.4	2068
Hb (g/L)	90–165	129	2122
Glucose (mmol/L)	2.28–9.48	4.86	2255
IGF-1 (μg/L)	7.3–1114	146.9	2227
IGFBP-3 (mg/L)	0.35–7.32	3.40	1478
FSH (mIU/mL)	0.06–11.47	3.01	306
LH (mIU/mL)	0.01–9.74	0.66	324
TES (ng/dL)	1.37–578.31	18.90	324
HDL-C (mmol/L)	0.83–1.75	1.35	29
LDL-C (mmol/L)	1.29–4.72	2.44	29
TC (mmol/L)	2.08–9.38	3.88	2086
TG (mmol/L)	0.38–2.28	0.97	31

Fe = iron, FSH = follicle-stimulating hormone, Hb = hemoglobin, HDL-C = high-density lipoprotein cholesterol, IGF-1 = insulin-like growth factor-1, IGFBP-3 = insulin-like growth factor binding protein 3, LDL-C = low-density lipoprotein cholesterol, LH = luteinizing hormone, TC = total cholesterol, TES = testosterone, TG = triglyceride, Zn = zinc.

### 3.2. The correlation between height and assay parameters

**Figure 3.**
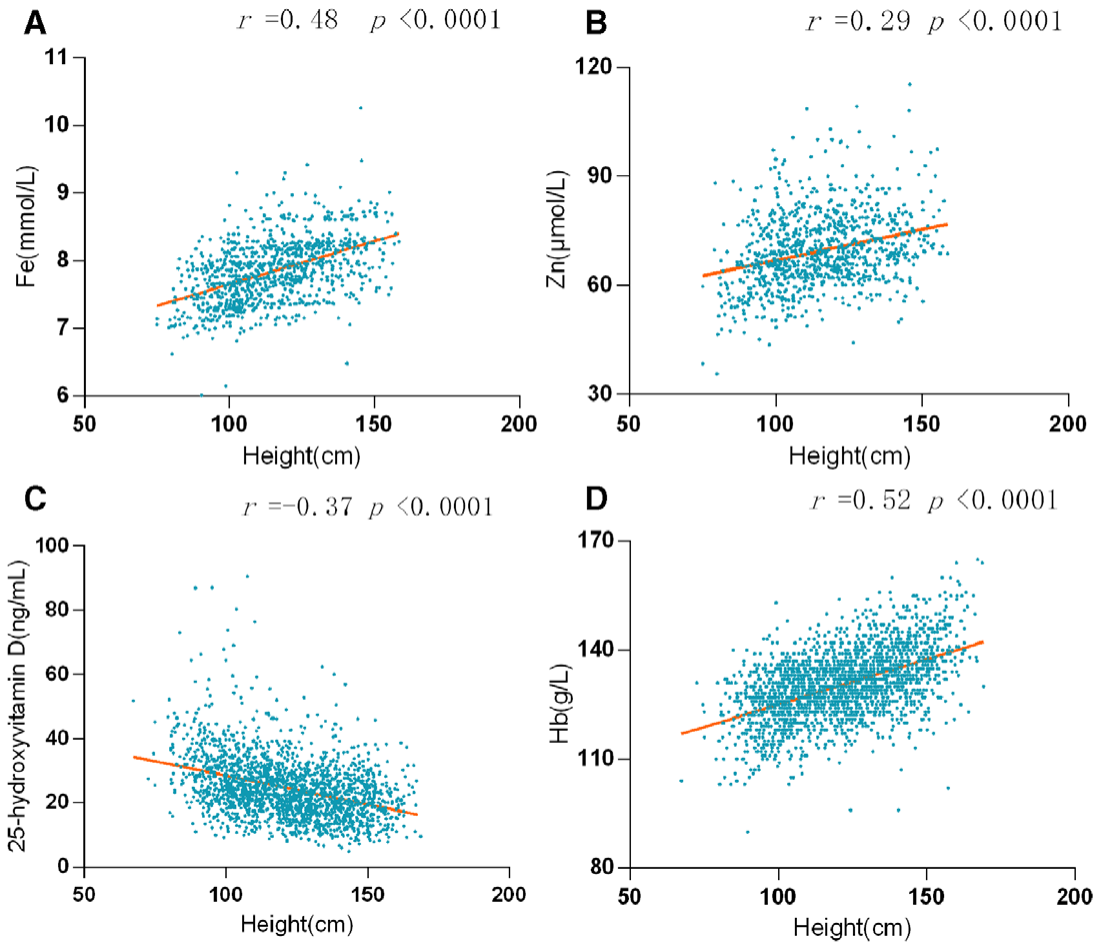
The connection between height and trace elements (A, B), 25-hydroxyvitamin D (C), and Hb (D). Hb = hemoglobin.

The research analyzed the connection between height and the concentrations of trace elements, 25-hydroxyvitamin D, and Hb (the result is shown in Fig. 3). Height was discovered to exhibit a somewhat positive association with Fe and Hb, and a weak association with Zn levels (Fe: *r* = 0.48, Hb: *r* = 0.52, Zn: *r* = 0.29, *P* < .0001, respectively). Conversely, a negative association was noted between height and 25-hydroxyvitamin D levels (*r* = −0.37, *P* < .0001).

**Figure 4.**
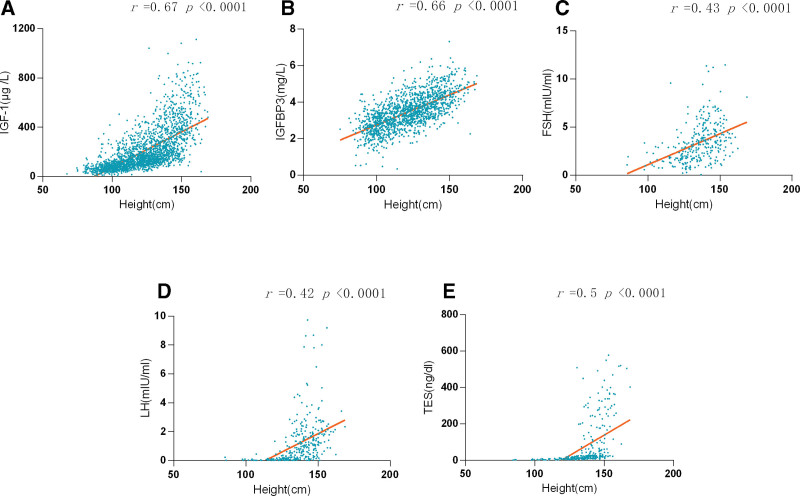
The relation between height and IGF-1 (A), IGFBP-3 (B), FSH (C), LH (D), and TES (E). FSH = follicle-stimulating hormone, IGF-1 = insulin-like growth factor-1, IGFBP-3 = insulin-like growth factor binding protein 3, LH = luteinizing hormone, TES = testosterone.

In addition, the research examined the relationship between stature and hormone concentration, particularly insulin-like growth factor-1 (IGF-1), insulin-like growth factor binding protein 3 (IGFBP-3), follicle-stimulating hormone (FSH), luteinizing hormone (LH), and testosterone (TES; as shown in Fig. 4). Significant associations were detected between height and IGF-1, IGFBP-3 levels (IGF-1: *r* = 0.67, *P* < .0001; IGFBP-3: *r* = 0.66, *P* < .0001). Moreover, moderate positive correlations were established between height and FSH, LH, and TES concentrations (FSH: *r* = 0.43, LH: *r* = 0.42, TES: *r* = 0.5, *P* < .0001, respectively).

**Figure 5.**
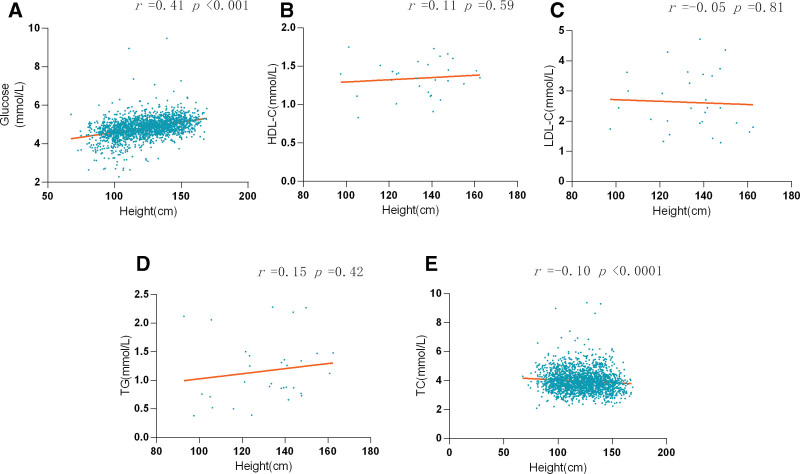
The correlation between height and glucose (A) and metabolism markers (B, C, D, E).

Finally, the analysis focused on the correlation between height and glucose as well as lipid metabolism markers, such as high-density lipoprotein cholesterol, low-density lipoprotein cholesterol, TC, and TG, as shown in Figure 5. A modest positive correlation was observed between height and glucose levels, with height demonstrating little to no association with lipid metabolism markers (glucose: *r* = 0.41, *P* < .0001; high-density lipoprotein cholesterol: *r* = 0.11, *P* = .59; low-density lipoprotein cholesterol: *r* = −0.05, *P* = .81; TG: *r* = 0.15, *P* = .42; TC: *r* = −0.1, *P* < .0001).

## 4. Discussion

This research analyzed the population characteristics of 2396 children diagnosed with short stature from our hospital. The investigation explored the correlation between height levels and the results of laboratory parameters. This study highlighted 3 primary clinical observations. First, there was a higher prevalence of short stature in boys compared with girls. Even though short children showed age-related increases in height and weight, these measurements did not meet the growth standards for average developing children. Second, the analysis uncovered a significant positive relationship between the height levels and IGF-1 and IGFBP-3. Additionally, there were moderate positive associations with Fe, Hb, glucose, and hormone indicators like FSH, LH, and TES, weak relationships with Zn, and limited connections with lipid metabolism indicators. Finally, a negative correlation was found between height and 25-hydroxyvitamin D levels.

With the rise in living standards, the height of children has generally seen an increase, though concerns about short stature persist.^[10,11]^ This research observed a higher prevalence of short stature in boys compared with girls, consistent with prior domestic studies.^[12]^ The analysis focused on the importance of trace elements in children’s growth and development,^[13,14]^ particularly examining the roles of Zn and Fe. It was found that Fe plays a more critical role in growth, likely due to its essential involvement in various metabolic processes like oxygen transport, DNA synthesis, and electron transport.^[15]^ Significantly, a strong association between height and Hb levels was established, underscoring the importance of Fe in growth. Some research has implied that weekly Fe supplementation may boost height,^[16]^ and anemia related to Fe utilization disorder has been associated with short stature.^[17]^ Inadequate levels of 25-hydroxyvitamin D have been shown to affect skeletal mineralization and bone growth during childhood, ultimately impactingadult height.^[18]^ This study uncovered a negative relationship between height and 25-hydroxyvitamin D levels, emphasizing the heightened physiological need for 25-hydroxyvitamin D in rapidly growing children. Notably, the rate of 25-hydroxyvitamin D supplementation declines notably with age in Chinese children,^[19]^ resulting in reduced serum 25-hydroxyvitamin D levels as height increases.

Regular somatic growth is a complex process that involves various genetic, nutritional, and hormonal elements that are closely linked. The axis controlling growth in children, known as the GH/IGF-1 axis, plays a key role during important developmental stages.^[20–22]^ IGF-1, which is similar to insulin in structure, acts on target organs directly to stimulate growth in response to GH.^[23]^ Irregular IGF-1 and IGFBP-3 secretion is commonly observed in many cases.^[24]^ IGFBP-3 acts as the primary transporter of IGF-1, reducing its free levels and prolonging its lifespan post-binding.^[25]^ This research found a strong positive connection between height and IGF-1 and IGFBP-3 in children with short stature, supporting prior knowledge and suggesting their potential utility as early screening markers. Furthermore, data indicate a link between IGF-1 and Hb levels in children with short stature,^[20]^ and it is expected to make new progress in the future. Numerous instances of reduced height in youngsters are linked to early maturation and initiation of the hypothalamic-pituitary-gonadal axis. Premature activation of gonadotropin-releasing hormone in the hypothalamus prompts early release of pituitary gonadotropins and sex hormones, initiating premature sexual organ maturation, heightened levels of sex hormones, early closure of growth plates, and ultimately impeding growth potential in children, leading to reduced stature in adults.^[11,26]^ Analysis of data reveals a significant increase in sex hormone levels once height surpasses around 130 cm, hinting at a possible influence on growth potential.

Through the examination of glucose and lipid metabolism data, this research revealed a modest positive association between height and blood glucose concentrations, with minimal correlation observed in blood lipid markers. A thorough assessment of prior studies showed limited support for a direct link between height and blood glucose as well as lipids. It is currently known that maintaining proper nutrition during childhood is crucial in safeguarding against growth and developmental issues caused by malnutrition.

Additionally, this research presents other shortcomings. For one thing, the examination of parental height’s influence on children’s stature remains incomplete. A predictive formula is available to gauge children’s ultimate height based on parental measurements,^[27]^ prompting the necessity for ongoing participant monitoring to delve deeper into this correlation. For another, the scarcity of lipid metabolism markers collected creates uncertainty about the study’s ability to precisely portray their interconnection. One plausible solution entails enlarging the sample size in forthcoming research endeavors and verifying outcomes through supplementary data acquisition.

## 5. Conclusion

Perhaps children with short stature are not frequently observed, however, studies indicate that they constitute a significant portion of the population. In this group, boys surpass girls in number, and both their weight and height show notable deviations from the established norms. Research data demonstrates a highly positive link between height and IGF-1 as well as IGFBP-3, a moderate positive association with Fe, Hb, glucose, and sex hormones, a weak positive correlation with Zn, a moderate negative correlation with 25-hydroxyvitamin D, and minimal correlation with lipid metabolism markers. Based on the findings of this large sample size study, these findings can provide valuable reference for parents when children undergo routine physical examination or when they are unwell and need blood tests. For example, it is essential to ensure the adequate consumption of vitamins and essential minerals, and seek prompt medical advice if hormone levels are found to be abnormal.

## Author contributions

**Funding acquisition:** Chuanwei Ban.

**Data curation:** Juan Wang, Qian Zeng.

**Formal analysis:** Ping Liu, Qingling Wang.

**Investigation:** Chuanwei Ban.

**Writing – original draft:** Chuanwei Ban.

**Writing – review & editing:** Xin Lv.
